# Women's Initial Experiences and up to a Year in Retrospect of Their Life Situation With a Confirmed Takotsubo Syndrome Diagnosis: A Single Case Study Literature Review

**DOI:** 10.1177/08980101211018343

**Published:** 2021-05-24

**Authors:** Bengt Fridlund, Eileen Bruteig, Rønnaug M. Dahlviken, Nina Fålun, Tone M. Norekvål

**Affiliations:** 60498 Haukeland University Hospital; 60496 Stavanger University Hospital; 96902 Oslo University Hospital; 60498 Haukeland University Hospital; 1658 University of Bergen; 60498 Haukeland University Hospital

**Keywords:** case study, holistic nursing, person-centered care, qualitative method, stressful life events, Takotsubo syndrome, women

## Abstract

**Purpose:** To describe women's initial experiences and up to a year in retrospect of their life situation with a confirmed Takotsubo syndrome (TS) diagnosis. **Method:** A single case study literature review based on nine articles published by nurses was analyzed deductively using a nursing life dimension model. **Results:** All but one case was conducted on the North American continent and TS had largely affected women with previous cardiac history and had been triggered by one or two stressful life events. The biophysical life dimension manifested in distinct and troublesome inconvenience and in retrospect in fretting and grievous ailments. The emotional dimension manifested in pronounced ways and attitude-related sensations, feelings or moods while the intellectual dimension manifested in an unmanageable world of thought. The spiritual–existential dimension manifested in a life-denying view of life and the socio-cultural dimension manifested in an asocial life. **Conclusions:** With TS best practice in mind and a person-centered care and holistic approach, comprehensive descriptions are needed of how women identify, interpret, and use knowledge to manage their life situation. Cardiac nurses need further comprehensive descriptions to implement actions. Prior to implementation of such programs, this knowledge needs to be disseminated among cardiac nurses and evaluated in international randomized controlled trials.

## Introduction

Takotsubo syndrome (TS), also commonly referred to as left ventricular apical ballooning syndrome, stress-induced cardiomyopathy, or broken heart syndrome, is a cardiac heart failure condition often triggered by acute major mental or physical stress (Lyon et al., 2016; Napp & Bauersachs, 2020; Sheppard, 2015; Y-Hassan & Tornvall, 2018). Stressful life events are defined as positive or negative distinct experiences that disrupt a person's usual activities, causing a substantial change and readjustment, such as the death of a loved one, divorce, marriage, illness or injury, and job changes (Cohen et al., 2019; Noone, 2017; Rahe & Arthur, 1978). Of those affected, 64–85% have recently experienced an acute period of grief, shock, or stress. The condition is either defined as a primary subtype where acute cardiac symptoms are the reason for seeking care or a secondary subtype made up of those already hospitalized for another medical, surgical, anesthesiologic, obstetric, or psychiatric condition (Lyon et al., 2016; Sheppard, 2015). However, there is a knowledge gap regarding gender and nature as well as the duration of the stressful life event (Gupta & Gupta, 2018; Nyman et al., 2019; Templin et al., 2015; Y-Hassan & Tornvall, 2018).

There are numerous theories related to the cause of this condition including direct catecholamine-mediated myocardial stunning, microvascular dysfunction, myocardial bridging and myocardial edema, left ventricular outflow tract obstruction, hormonal and genetic factors, role of hypovolemia, hyponatremia and syndrome of inappropriate hypersecretion, malignancy and carbohydrate antigen, and systematic inflammation or anatomical variations, such as a smaller left ventricle (Gupta & Gupta, 2018; Kato et al., 2017; Napp & Bauersachs, 2020; Sheppard, 2015).

The clinical picture ranges from hemodynamically stable to the development of cardiogenic shock (15–20%), pulmonary edema (20–25%), ventricular tachycardia (8–10%) and, in the worst case, death (0.67–4%) (Ghadri et al., 2018a; Gupta & Gupta, 2018; Kato et al., 2017; Lyon et al., 2016). The condition, characterized by a transient hypokinesis of the left ventricular apex, produces many symptoms similar to acute coronary syndrome (ACS) (Ghadri et al., 2018a; Y-Hassan & Tornvall, 2018), such as chest pain, electrocardiography (ECG) changes, and moderately elevated cardiac biomarkers. Up to 10% of ACS admissions are possibly due to TS (Daniel et al., 2015). The condition requires emergency treatment, including coronary angiography, to distinguish it from ACS (Ghadri et al., 2018b; Tornvall et al., 2016). In most cases, TS is completely reversible if adequate heart failure treatment is implemented, and the prognosis is good after surviving the acute phase (Ghadri et al., 2018b; Gupta & Gupta, 2018; Yoshikawa, 2015). Outside the hospital however, follow-up is still scarce, especially with respect to self-reports by persons with confirmed TS diagnosis, and differs greatly with regard to long-term prognosis (Lyon et al., 2016; Sundelin et al., 2020; Yoshikawa, 2015).

The first 30 years of knowledge on TS from a medical patient-centered care perspective (Dote et al., 1991) show that today's best TS practice is based on consensus and position statement papers (Ghadri et al., 2018a, 2018b; Lyon et al., 2016), but guidelines have yet to be developed. At the same time, the best practice from a nursing person-centered perspective (Ekman et al., 2011; Santana et al., 2018), including the person's own statements of reasons, feelings, and needs (Curragh et al., 2020) in order to establish a partnership followed by shared decision making and understanding (Morgan & Yoder, 2012), is only based on five empirical Scandinavian TS studies with an explorative approach (Dahlviken et al., 2015; Mäenpää et al., 2020; Sundelin et al., 2020; Wallström et al., 2016a, 2016b). A case study that includes a person-centered and holistic approach to a person's life is another way of exploring close, in-depth, and detailed patient-reported outcomes and knowledge in a defined context to generate and illustrate theories and models that describe how different aspects of a person's life interact (Ebneyamini & Sadeghi Moghadam, 2018; Stjelja, 2013; Yin, 2009). The person is his/her own expert on the body, mind, and spirit, while best practice managed by cardiac nurses is based on a partnership built on statements representing interaction (Curragh et al., 2020). This is a prerequisite for providing person-centered care to a person with a confirmed TS diagnosis, particularly after a hospital stay, in order to ensure sound recovery and preventive measures (Mäenpää et al., 2020; Sundelin et al., 2020; Wallström et al., 2016b, 2019).

### Theoretical Model

According to Sarvimäki and Stenbock-Hult’s (1992) life situation model, a person expresses and lives through five dimensions: biophysical, emotional, intellectual, spiritual–existential, and socio-cultural dimensions of life. The biophysical dimension consists of various systems for maintaining life processes, such as respiratory, excretory, digestive, and immune system. Physical illness manifests as suffering comprising impaired physical functions in this dimension. Under the emotional dimension, the person expresses how they view themselves and the world through affective states, emotions, and moods. The intellectual dimension describes how a person perceives realities, and identifies and interprets the knowledge they are given to deal with the situation they find themselves in. The norms, ideals, and values that guide a person's life are part of the spiritual–existential dimension. The socio-cultural dimension deals with a person's interpersonal relationships, such as interaction with family, school, and work.

Cultural factors such as ethnic groups, traditions, and what is customary are also covered here (Sarvimäki & Stenbock-Hult, 1992). The five dimensions—each described to clinically capture the entire person—aim to present and understand a person as a whole, but the whole is more than the sum of its parts. The processes in each dimension also impact each other, which makes it challenging to place a phenomenon in only one dimension (Sarvimäki & Stenbock-Hult, 1992). Accordingly, cardiac nurses who practice person-centered care also practice a holistic approach to the person's life, that is considering the person from all aspects or dimensions of life and their interaction (Bergtun et al., 2019; Nordblom et al., 2017). Accordingly, the purpose of this study was to describe women's initial experiences and up to a year in retrospect of their life situation with a confirmed TS diagnosis.

## Method

### Design

The design used was a single case study literature review (Yin, 2009) carried out using a deductive method based on Sarvimäki and Stenbock-Hult’s (1992) life situation model and conducted as a qualitative content analysis at the manifest level (Graneheim & Lundman, 2004).

### Inclusion and Exclusion Criteria

Inclusion criteria were articles containing statements by females with a confirmed TS diagnosis up to a year in retrospect. The articles were published by nurses in English-language peer-reviewed nursing journals until 2019. Exclusion criteria were women with cognitive impairment that is unable to properly and reliably describe their manifestations and articles containing statements published in medical journals, that is in order to avoid the medical perspective in favor of the nursing perspective.

#### Literature Search

MeSH terms were used to find the correct search term. Searches were performed for one keyword at a time and subsequently combined, in order to generate as many articles as possible. Based on the inclusion and exclusion criteria, literature searches were performed in the databases Cinahl and PubMed with combinations of the keywords “takotsubo syndrome” AND “female” AND “nursing” AND “case.” Searches were also performed for “woman,” and “gender” as well as “broken heart syndrome,” “left ventricular apical ballooning syndrome,” “stress-induced cardiomyopathy,” and “takotsubo cardiomyopathy,” but yielded no further hits. Due to few relevant hits, searches were also performed in WorldCat using the same search strategy, as well as hand searches.

#### Selection

**Figure 1.**
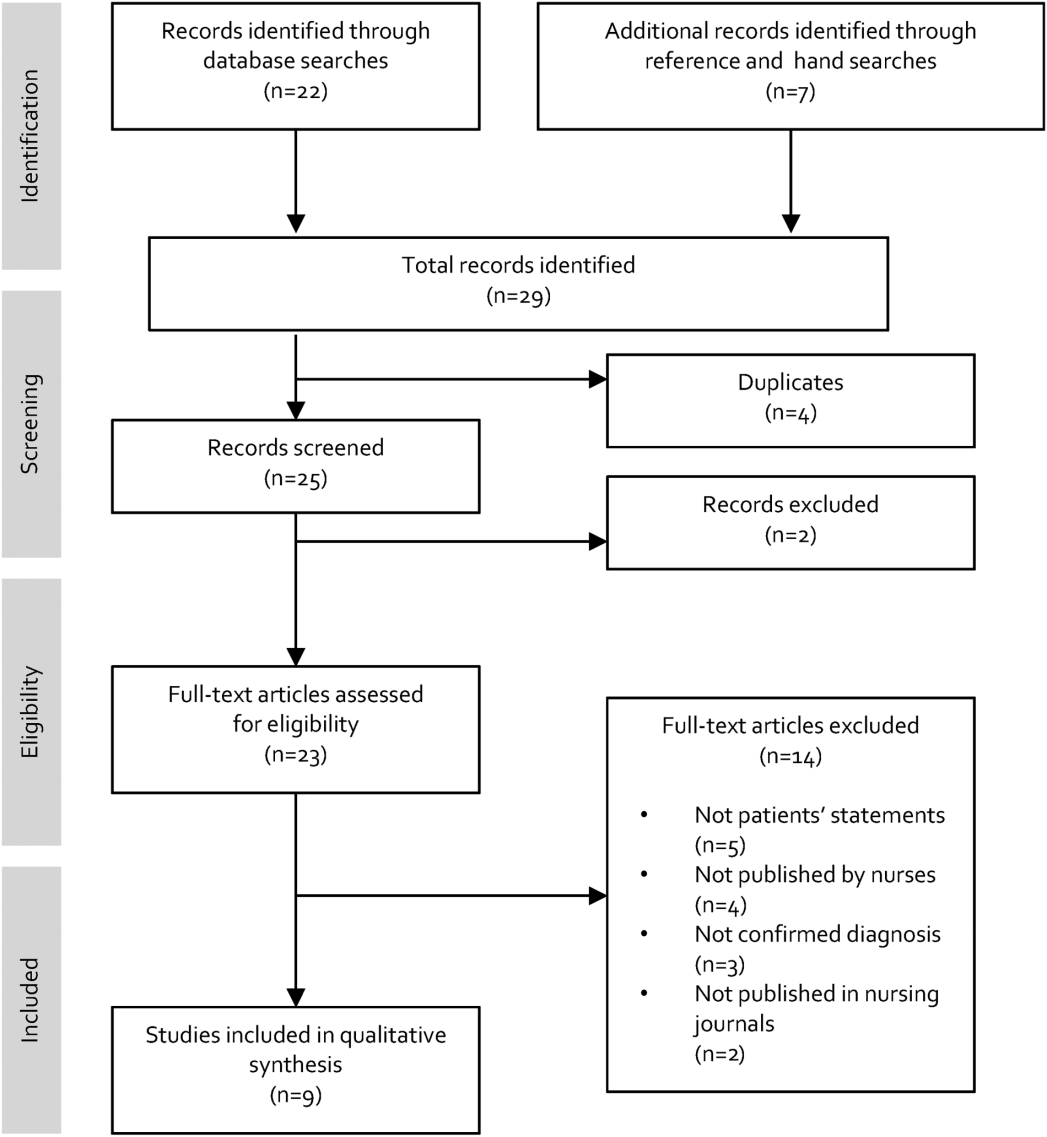
Flow diagram of data selection and quality assessment process based on the Preferred Reporting Items for Systematic Reviews and Meta-Analyses (PRISMA statement) (Liberati et al., 2009).

Articles were selected based on the inclusion and exclusion criteria. Several articles were excluded because they were not relevant to the purpose as they did not contain patients' statements, were not conducted by a nurse, or were published in a nursing journal. This search strategy generated a total of seven articles in PubMed, four in Cinahl, and 22 in WorldCat. Of these, four were duplicates, which brought the total number in WorldCat to 18. Another seven were found through the hand search based on the references from derived articles. However, two articles were not full-text written in English, meaning a total of 23 articles were screened for and eligible for quality assessment (Figure 1).

#### Quality Assessment

In accordance with Streit Olness et al. (2005), all articles were carefully read to assess the quality of the social validity of the individual clinical statements. Specifically, the focus was on evaluating researchers and clinicians' analytic and holistic assessment of the statements' quality, and overall, the characteristics of patient statements along a continuum of quality.

A total of 14 articles were excluded, the majority of which consisted of statements not including the patient's own statements of reasons, feelings, and needs, but more pure Latin or medical terms implying that they were created by healthcare professionals.

After completing the quality assessment, the final outcome was nine articles including five from WorldCat and another four from the hand search.

#### Ethical Assessments

It was important to bear the four ethical principles, autonomy, justice, charity, and non-harm (Council for International Organizations of Medical Sciences, 2002), in mind in relation to both criteria selection and quality assessment of articles. The principle of justice is particularly important, since researchers have an ethical obligation to treat each person in accordance with what is morally right and proper (Council for International Organizations of Medical Sciences, 2002).

#### Data Analysis

A qualitative content analysis at a manifest level (Graneheim & Lundman, 2004) was deductively performed according to Sarvimäki and Stenbock-Hult’s (1992) life situation model by the research team, whose members possess a high level of expertise in both the topic and method, also bearing in mind the significance of preunderstanding in the analysis procedure. Due to the quality of content of each article to be analyzed, each had to be analyzed at a manifest level to provide a credible finding, which was also expected. Each article was carefully read and the subjective–personal content was identified and determined by research team consensus on context, and coded and categorized according to characteristics, that is body, mind, and spirit manifestations based on the person's statements (Graneheim & Lundman, 2004).

## Findings

### Socio-Demographic and Context Characteristics

All but one case was conducted on the North American continent. The women were largely postmenopausal, but the age span (32–82 years) also involved younger women. It was common for the women to have previous cardiac history, and especially experience of a stressful life event. The stressful life event was of a physical or emotional nature or both, and was either prolonged, fairly recent, just prior, or sudden in relation to the initial manifestations.

### Biophysical Dimension Manifesting in Distinct and Troublesome Inconvenience and in Retrospect in Fretting and Grievous Ailments (Table 1)

*During the initial manifestations*, the physically distinct and troublesome inconvenience was mostly related to the heart (chest pain and palpitations), brain (dizziness and syncope), lungs (breathlessness and dry cough), and stomach (sense of a lump, upset). Another manifestation was a general malaise, comprising affected peripheral blood circulation (cold, paleness, thirst, and nasal bleeding). *Up to a year in retrospect, manifestations* were still related to the heart (episodes of palpitations), lungs (breathlessness) and stomach (sense of a lump, upset), and also with general tiredness (fatigue, lower back pain, weak-voiced) in fretting and grievous ailments.

**Table 1.** Descriptions of the Socio-demographic and Situational Characteristics and the Manifest Deductive Data Analysis According to Sarvimäki and Stenbock-Hult’s (1992) Five Life Dimensions Model of the Nine Women's Initial Experiences and up to a Year in Retrospect With a Confirmed Takotsubo Syndrome Diagnosis.

Reference	Setting, age, and cardiac history	Stressful life event trigger in a time perspective	Biophysical dimension Initial manifestation, and *a year in retrospect*	Emotional dimension Initial manifestation, and *a year in retrospect*	Intellectual dimension Initial manifestation, and *a year in retrospect*	Spiritual–existential dimension Initial manifestation, and *a year in retrospect*	Socio-cultural dimension Initial manifestation, and *a year in retrospect*
Baxter (2008)	U.S.A., 58 years and cardiac history	Ongoing bad work environment	Cough, speaking in short clipped word phrases	Anxious, crying, panic attack	Overwork, stressful work	—	—
Brenner and Powers (2008)	U.S.A., 77 years and cardiac history	Recently widowed	Malaise last 2-3 weeks, palpitations 2-3 hr every 2-3 weeks the last months	—	—	—	—
Granitto et al. (2010)	U.S.A., 67 years and cardiac history	Son died recently in motorcycle accident	Chest pain, shortness of breath	Anxious	—	—	—
Hanson (2008)	U.S.A., 79 years and cardiac history	Prior intense religious discussion; husband passed away recently	Chest pain, diaphoresis, dizziness, nausea, palpitations, reddened face	Excitement	—	—	—
Nyeche and Winokur (2017)	U.S.A., 48 years and no cardiac history	Son recently died in motorcycle accident	Chest pain, shortness of breath	Grief, sadness	—	Suicidal thoughts	—
Pfister et al. (2010)	USA, 31 years and no cardiac history	Prior major surgical procedure	Ashen and pale, cold and thirsty, fast heartbeats	—	—	—	—
Swenson et al. (2018)	U.S.A., 71 years and cardiac history	Prior major surgical procedure; son recently diagnosed with severe cancer	Radiating chest pain	—	—	—	—
Therkleson and Stronach (2015)	New Zealand, 82 years and no cardiac history	Recently frequent earthquake aftershocks with damaged home and social isolation	Fast heartbeats, nose bleeds, paleness, shortness of breath, stomach ache *A year in retrospect:* *Brittle and weak voice, episodes of fast heartbeats, fatigue, low back pain, shortness of breath, stomach ache*	Exhaustion *A year in retrospect: anxious, exhaustion, nightmares, panic attacks*	— *A year in retrospect: lack of energy and powerlessness, general body tension, general wear and tear, scattered thinking*	— *A year in retrospect: lost faith in the future, lost self-confidence, lost self-knowledge, sense of foreboding*	Broken relationships, socially isolated *A year in retrospect: socially isolated, social inaction*
Turris (2006)	Canada, 66 yrs and cardiac history	Suddenly when running in a parking lot trying to locate the car	Chest pain, dizziness, shortness of breath	—	—	—	—

### Emotional Dimension Manifesting in Pronounced Ways- and Attitude-Related Sensations, Feelings, or Moods (Table 1)

*During the initial manifestations* as well as *Up to a year in retrospect,* the emotions were pronounced as sudden (panic attack) or more persistent (exhaustion) and either as extroverted (crying and excitement) or introverted (sadness and grief) emotions, from sensations, to feelings and moods.

### Intellectual Dimension Manifesting in an Unmanageable World of Thought (Table 1)

*During the initial manifestations,* the world of thought was unmanageable due to a stressful environment. *Up to a year in retrospect,* this stress-induced situation had evolved into a powerlessness and persistent unmanageable world of thought effectuating in scattered thinking.

### Spiritual–Existential Dimension Manifesting in a Life-Denying View of Life (Table 1)

*During the initial manifestations,* gloomy and dark thoughts had evolved into a negative ethos. *Up to a year in retrospect,* this negative ethos was confirmed as a loss of self-confidence as well as faith in the future.

### Socio-Cultural Dimension Manifesting in an Asocial Life (Table 1)

*During the initial manifestations* as well as *up to a year in retrospect,* a passivity or inability to perform social activities led to the loss of necessary relationships thereby engendering social isolation.

## Discussion

The wide age range of women with a confirmed TS diagnosis confirms what is already known (Napp & Bauersachs, 2020; Sheppard, 2015; Y-Hassan & Tornvall, 2018). However, nurses should bear in mind that young healthy persons are also potential TS candidates given that ACS condition mimicking occurs (Gupta & Gupta, 2018). In all women, TS onset had been preceded by a stressful life event of a physical and emotional nature, in line with what is already known (Nyman et al., 2019), but it also became evident that two stressful life events had triggered the TS—in a two-phase interaction—one of a more chronic or long-term nature and the other of a more acute or sudden nature (Cohen et al., 2019).

In a person-centered and holistic approach to a person's life situation, all the manifestations were very tangible and extensive, and many alluded to the biophysical and emotional life dimensions, but few to the other three dimensions. This is remarkable as a stressful life event in human existence probably finds expressions in other manifestations as well. Plausible reasons are that cardiac nurses or teams do not ask the type of existential questions that it is natural and important to ask when practicing a person-centered care philosophy (Ekman et al., 2011; Wallström et al., 2016a), suggesting an educational upgrade is required.

The biophysical dimension manifested as distinct and troublesome inconvenience and in retrospect in fretting and grievous ailments. This implies that the root cause remains but takes on expressions of more chronic and general manifestations, such as fatigue and powerlessness, demonstrating the need for secondary preventive care follow-up (Sundelin et al., 2020; Wallström et al., 2016a).

A similar phenomenon is observed regarding the emotional manifestations, as no concrete changes or improvements are seen in retrospect, which must again be taken into consideration with regard to secondary preventive care aspects. The two emotional attitudes—introversion and extroversion (Segerstrom, 2000) are of interest for the same reason, providing a plausible explanation for why cardiac nurses and teams perhaps misunderstand the introvert manifestations, which also support the need for an educational upgrade.

The intellectual dimension manifested in an unmanageable world of thought showed—in line with the biophysical and emotional dimensions—no life improvements. However, the condition initially presented during stressful work which, in retrospect, effectuated a general state of tension expressed as a lack of energy and feeling of powerlessness. This is in line with both Sundelin et al. (2020) and Wallström et al. (2016a) as well as being established in the American and European guidelines (van der Meer et al., 2019).

Not surprisingly, the spiritual–existential dimension manifested in a life-denying view and suicidal thoughts. A loss of faith in the future was confirmed as a subsequent loss of self-knowledge and self-confidence. Medical follow-up after a few months often showed sufficient return of systolic function with normal ejection fraction, and accordingly positive results from a medical-patient care perspective. However, follow-up from a nursing person-centered perspective comprising secondary preventive care in general, and health promotion care in particular, is still inexplicably lacking (Mäenpää et al., 2020; Sundelin et al., 2020; Wallström et al., 2019).

Not surprisingly, but in line with the life dimensions previously reflected, the social–cultural dimension presented a passivity or inability to perform social activities resulting in lost relationships and thereby engendering social isolation, especially in older widows, which in itself is a potent cardiovascular risk factor (Valtorta et al., 2016). It is obviously not easy to examine isolation if there is no follow-up care, and especially if the person receives the biomedical information that the heart “is fit again.”

However, from a nursing person-centered perspective with a holistic approach (Ekman et al., 2011; Santana et al., 2018; Sarvimäki & Stenbock-Hult, 1992), the presentation in manifestations of reason, will, feelings, and needs (Curragh et al., 2020) shows an obviously sensitive and powerless elderly woman whose entire life situation is unmanageable and in complete imbalance (Dahlviken et al., 2015; Sundelin et al., 2020; Wallström et al., 2016a). Accordingly, if no such information exists it is difficult for a cardiac nurse or team to be able to assess what knowledge the person needs and further, to know how to help her understand the situation and make best practice healthcare choices in line with suggested health literacy actions (Mäenpää et al., 2020; Sørensen et al., 2018).

### Methodological Considerations

The case study review was chosen as it is a preferred design when focusing on exploring new areas with a limited theoretical background as well as describing processes, effects, and circumstances of a condition (Yin, 2009).

Moreover, the single case study design was chosen due to its collection and analysis merits. This applies regardless of the nature of the case, be it a representative, typical case or a unique, extreme case, as well as for longitudinal cases following what happens over time or revelatory cases that have not previously been explored (Ebneyamini & Sadeghi Moghadam, 2018; Stjelja, 2013). All these types of cases were assumed to exist and also found in the data collection and deductively analyzed according to Sarvimäki and Stenbock-Hult’s (1992) life situation model. One of three preferred approaches to explaining the phenomenon, that is the explanatory case study design, was also used in order to provide a detailed description of the facts of the case. This was followed by reflection on reasonable manifestations congruent with the facts described. Inclusion and exclusion criteria based on MeSH terms were defined to maintain a nursing person-centered perspective ensuring a credible selection of studies.

Similarly, the use of a well-proven quality assessment form (Streit Olness et al., 2005) contributed to ensuring the social validity of the individual clinical statements. During the quality assessment and data analysis processes, the research team was conscious of the group's preunderstanding and made no assumptions without reviewing and analyzing the studies until consensus was reached.

All but one study was conducted on the North American continent, which probably represents a limitation in transferability due to socio-cultural and socio-economic differences in other continents. Another limitation concerns the relatively low number of available and credible cases, which also affects data access and thus the breadth and depth of the analysis, thereby reducing the outcome scope.

## Conclusions and Implications

There is a lack of TS best practice in single case studies from a nursing person-centered perspective, but a severe life situation is demonstrated in older women both initially and up to a year in retrospect. The triggers were physical and emotional in nature, but there was also less recognition of the triggers, and of both their acute and long-term nature. In addition to the commonly accepted physical and emotional manifestations, there were also intellectual, spiritual–existential, and socio-cultural manifestations, which are less recognized either initially or up to a year in retrospect. Cardiac nurses and teams need further comprehensive descriptions of women's life situations both initially and up to a year in retrospect in order to develop and implement actions for women with a confirmed TS diagnosis. Before such programs and guidelines are communicated and implemented, randomized controlled trials on different continents need to be performed and results disseminated among cardiac nurses and teams.

## References

[bibr1-08980101211018343] Baxter G. L. (2008, April). A 58-year-old woman with stress-induced cardiomyopathy (Takotsubo). Journal of Emergency Nursing, 34(2), 134-136. 10.1016/j.jen.2007.05.003 18358352

[bibr2-08980101211018343] Bergtun S. Oterhals K. Fridlund B. (2019, January). Patients’ experiences 1–6 months after atrial fibrillation ablation: An holistic perspective. Journal of Advanced Nursing, 75(1), 150-160. 10.1111/jan.13843 30187542

[bibr3-08980101211018343] Brenner Z. R. Powers J. (2008, January-February). Takotsubo cardiomyopathy. Heart & Lung, 37(1), 1-7. 10.1016/j.hrtlng.2006.12.003 18206521

[bibr4-08980101211018343] Cohen S. Murphy M. L. M. Prather A. A. (2019, January 4). Ten surprising facts about stressful life events and disease risk. Annual Review of Psychology, 70, 577-597. 10.1146/annurev-psych-010418-102857 PMC6996482 29949726

[bibr5-08980101211018343] Council for International Organizations of Medical Sciences (2002). International ethical guidelines for biomedical research involving human subjects. World Health Organization. http://www.cioms.ch/publications/guidelines/guidelines_nov_2002_blurb.htm 14983848

[bibr6-08980101211018343] Curragh C. Rein M. Green G. (2020, January). Takotsubo syndrome: Voices to be heard. European Journal of Cardiovascular Nursing, 19(1), 4-7. 10.1177/1474515119886078 31686533

[bibr7-08980101211018343] Dahlviken R. M. Fridlund B. Mathisen L. (2015, June). Women’s experiences of Takotsubo cardiomyopathy in a short-term perspective–a qualitative content analysis. Scandinavian Journal of Caring Sciences, 29(2), 258-267. 10.1111/scs.12158 24953349

[bibr8-08980101211018343] Daniel M. Ekenbäck C. Agewall S. Brolin E. B. Caidahl K. Cederlund K. Collste O. Eurenius L. Frick M. Younis-Hassan S. Henareh L. Jernberg T. Malmqvist K. Spaak J. Sörensson P. Hofman-Bang C. Tornvall P. (2015, September 15). Risk factors and markers for acute myocardial infarction with angiographically normal coronary arteries. American Journal of Cardiology, 116(6), 838-844. 10.1016/j.amjcard.2015.06.011 26251000

[bibr9-08980101211018343] Dote K. Sato H. Tateishi H. Uchida T. Ishihara M. (1991). Myocardial stunning due to simultaneous multivessel coronary spasms: A review of 5 cases. Journal of Cardiology, 21(2), 203-214. https://www.ncbi.nlm.nih.gov/pubmed/1841907 1841907

[bibr10-08980101211018343] Ebneyamini S. Sadeghi Moghadam M. R. (2018, December). Toward developing a framework for conducting case study research. International Journal of Qualitative Methods, 17(1), 1-11. 10.1177/1609406918817954

[bibr11-08980101211018343] Ekman I. Swedberg K. Taft C. Lindseth A. Norberg A. Brink E. Carlsson J. Dahlin-Ivanoff S. Johansson I. L. Kjellgren K. Liden E. Öhlen J. Olsson L. E. Rosén H. Rydmark M. Sunnerhagen K. S. (2011, December). Person-centered care–ready for prime time. European Journal of Cardiovascular Nursing, 10(4), 248-251. 10.1016/j.ejcnurse.2011.06.008 21764386

[bibr12-08980101211018343] Ghadri J. R. Wittstein I. S. Prasad A. Sharkey S. Dote K. Akashi Y. J. Cammann V. L. Crea F. Galiuto L. Desmet W. Yoshida T. Manfredini R. Eitel I. Kosuge M. Nef H. M. Deshmukh A. Lerman A. Bossone E. Citro R. Templin C. (2018a, June 7). International expert consensus document on takotsubo syndrome (part I): Clinical characteristics, diagnostic criteria, and pathophysiology. European Heart Journal, 39(22), 2032-2046. 10.1093/eurheartj/ehy076 29850871 PMC5991216

[bibr13-08980101211018343] Ghadri J. R. Wittstein I. S. Prasad A. Sharkey S. Dote K. Akashi Y. J. Cammann V. L. Crea F. Galiuto L. Desmet W. Yoshida T. Manfredini R. Eitel I. Kosuge M. Nef H. M. Deshmukh A. Lerman A. Bossone E. Citro R. Templin C. (2018b, June 7). International expert consensus document on Takotsubo syndrome (part II): Diagnostic workup, outcome, and management. European Heart Journal, 39(22), 2047-2062. 10.1093/eurheartj/ehy077 29850820 PMC5991205

[bibr14-08980101211018343] Graneheim U. H. Lundman B. (2004, February). Qualitative content analysis in nursing research: Concepts, procedures and measures to achieve trustworthiness. Nurse Education Today, 24(2), 105-112. 10.1016/j.nedt.2003.10.001 14769454

[bibr15-08980101211018343] Granitto M. H. Norton C. K. Sher R. Baldia C. (2010). Takotsubo cardiomyopathy: Implications for nursing practice. Advanced Emergency Nursing Journal, 32(1), 83-91. 10.1097/TME.0b013e3181cb75b6

[bibr16-08980101211018343] Gupta S. Gupta M. M. (2018). Takotsubo syndrome. Indian Heart Journal, 70(1), 165-174. 10.1016/j.ihj.2017.09.005 29455773 PMC5902911

[bibr17-08980101211018343] Hanson T. (2008). Takotsubo cardiomyopathy: A case study of stress induced transient left ventricular apical ballooning syndrome. The Internet Journal of Advanced Nursing Practice, 10, 1-8. https://ispub.com/IJANP/10/2/6269

[bibr18-08980101211018343] Kato K. Lyon A. R. Ghadri J. R. Templin C. (2017, September). Takotsubo syndrome: Aetiology, presentation and treatment. Heart (British Cardiac Society), 103(18), 1461-1469. 10.1136/heartjnl-2016-309783 28839096

[bibr19-08980101211018343] Liberati A. Altman D. G. Tetzlaff J. Mulrow C. Gøtzsche P. C. Ioannidis J. P. A. Clarke M. Devereaux P. J. Kleijnen J. Moher D. (2009). The PRISMA statement for reporting systematic reviews and meta-analyses of studies that evaluate healthcare interventions: Explanation and elaboration. BMJ, 339, b2700. 10.1136/bmj.b2700 19622552 PMC2714672

[bibr20-08980101211018343] Lyon A. R. Bossone E. Schneider B. Sechtem U. Citro R. Underwood S. R. Sheppard M. N. Figtree G. A. Parodi G. Akashi Y. J. Ruschitzka F. Filippatos G. Mebazaa A. Omerovic E. (2016, January). Current state of knowledge on takotsubo syndrome: A position statement from the taskforce on takotsubo syndrome of the heart failure association of the european society of cardiology. European Journal of Heart Failure, 18(1), 8-27. 10.1002/ejhf.424 26548803

[bibr21-08980101211018343] Morgan S. Yoder L. H. (2012). A concept analysis of person-centered care. Journal of Holistic Nursing, 30(1), 6-15. 10.1177/0898010111412189 21772048

[bibr22-08980101211018343] Mäenpää S. Ekstrand E. Petersson C. Nymark C. (2020, August 17). Patients’ experiences when afflicted by Takotsubo syndrome – is it time for guidelines? Scandinavian Journal of Caring Sciences. 10.1111/scs.12897 32808352

[bibr23-08980101211018343] Napp L. C. Bauersachs J. (2020, May). Takotsubo syndrome: Between evidence, myths, and misunderstandings. Herz, 45(3), 252-266. 10.1007/s00059-020-04906-2 32206851 PMC7198647

[bibr24-08980101211018343] Noone P. A. (2017, October). The Holmes-Rahe stress inventory. Occupational Medicine, 67(7), 581-582. 10.1093/occmed/kqx099 29048597

[bibr25-08980101211018343] Nordblom A.-K. Broström A. Fridlund B. (2017). Impact on a person’s daily life during episodes of supraventricular tachycardia: A qualitative content analysis from a holistic perspective. Journal of Holistic Nursing, 35(1), 33-43. 10.1177/0898010116639722 27004745

[bibr26-08980101211018343] Nyeche M. N. Winokur E. J. (2017, September). Caring for the Takotsubo cardiomyopathy patient. The Journal for Nurse Practitioners, 13(9), 635-641. 10.1016/j.nurpra.2017.07.024

[bibr27-08980101211018343] Nyman E. Mattsson E. Tornvall P. (2019, May). Trigger factors in Takotsubo syndrome – a systematic review of case reports. European Journal of Internal Medicine, 63, 62-68. 10.1016/j.ejim.2019.02.017 30833207

[bibr28-08980101211018343] Pfister S. Wagar P. Casserly I. P. (2010, October). Stress-related cardiomyopathy in a 31-year-old woman. AANA Journal, 78(5), 406-411. https://www.ncbi.nlm.nih.gov/pubmed/21067089 21067089

[bibr29-08980101211018343] Rahe R. H. Arthur R. J. (1978). Life change and illness studies: Past history and future directions. Journal of Human Stress, 4(1), 3-15. 10.1080/0097840X.1978.9934972 346993

[bibr30-08980101211018343] Santana M. J. Manalili K. Jolley R. J. Zelinsky S. Quan H. Lu M. (2018, April). How to practice person-centred care: A conceptual framework. Health Expectations, 21(2), 429-440. 10.1111/hex.12640 29151269 PMC5867327

[bibr31-08980101211018343] Sarvimäki A. Stenbock-Hult B. (1992). Caring: An introduction to health care from a humanistic perspective. Foundation for Nursing Education.

[bibr32-08980101211018343] Segerstrom S. C. (2000, Summer). Personality and the immune system: Models, methods, and mechanisms. Annals of Behavioral Medicine, 22(3), 180-190. 10.1007/BF02895112 11126462

[bibr33-08980101211018343] Sheppard M. N. (2015, December). Takotsubo syndrome – stress-induced heart failure syndrome. European Cardiology Review, 10(2), 83-88. 10.15420/ecr.2015.10.2.83 30310431 PMC6159416

[bibr34-08980101211018343] Stjelja M. (2013). The case study approach: Some theoretical, methodological and applied considerations. Australian Government, Department of Defence, Defence Science and Technology Organisation. https://apps.dtic.mil/dtic/tr/fulltext/u2/a588465.pdf

[bibr35-08980101211018343] Streit Olness G. Ulatowska H. Carpenter C. Williams-Hubbard L. Dykes J. (2005, August). Holistic assessment of narrative quality: A social validation study. Aphasiology, 19(3-5), 251-262. 10.1080/02687030444000723 PMC12393826 40893410

[bibr36-08980101211018343] Sundelin R. Bergsten C. Tornvall P. Lynga P. (2020, June). Self-rated stress and experience in patients with Takotsubo syndrome: A mixed methods study. European Journal of Cardiovascular Nursing, 1474515120919387. 10.1177/1474515120919387 PMC7817986 32491953

[bibr37-08980101211018343] Swenson S. Bull J. Chen I. B. Joseph D. Joseph J. Varghese M. Dellostritto R. A. (2018, July). Takotsubo cardiomyopathy: A discussion and case study. Journal of the American Association of Nurse Practitioners, 30(7), 392-397. 10.1097/JXX.0000000000000078 29979298

[bibr38-08980101211018343] Sørensen K. Karuranga S. Denysiuk E. McLernon L. (2018, December). Health literacy and social change: Exploring networks and interests groups shaping the rising global health literacy movement. Global Health Promotion, 25(4), 89-92. 10.1177/1757975918798366 30444170

[bibr39-08980101211018343] Templin C. Ghadri J. R. Diekmann J. Napp L. C. Bataiosu D. R. Jaguszewski M. Cammann V. L. Sarcon A. Geyer V. Neumann C. A. Seifert B. Hellermann J. Schwyzer M. Eisenhardt K. Jenewein J. Franke J. Katus H. A. Burgdorf C. Schunkert H. Luscher T. F. (2015, September). Clinical features and outcomes of takotsubo (stress) cardiomyopathy. New England Journal of Medicine, 373(10), 929-938. 10.1056/NEJMoa1406761 26332547

[bibr40-08980101211018343] Therkleson T. Stronach S. (2015, December). Broken heart syndrome: A typical case. Journal of Holistic Nursing, 33(4), 345-350. 10.1177/0898010115569883 25673580 PMC4647181

[bibr41-08980101211018343] Tornvall P. Collste O. Ehrenborg E. Jarnbert-Petterson H. (2016, April). A case-control study of risk markers and mortality in Takotsubo stress cardiomyopathy. Journal of the American College of Cardiology, 67(16), 1931-1936. 10.1016/j.jacc.2016.02.029 27102508

[bibr42-08980101211018343] Turris S. A. (2006, August). A 66-year-old woman with Takotsubo syndrome. Journal of Emergency Nursing, 32(4), 313-316. 10.1016/j.jen.2006.04.009 16863878

[bibr43-08980101211018343] Valtorta N. K. Kanaan M. Gilbody S. Ronzi S. Hanratty B. (2016, July). Loneliness and social isolation as risk factors for coronary heart disease and stroke: Systematic review and meta-analysis of longitudinal observational studies. Heart (British Cardiac Society), 102(13), 1009-1016. 10.1136/heartjnl-2015-308790 27091846 PMC4941172

[bibr44-08980101211018343] van der Meer P. Gaggin H. K. Dec G. W. (2019, June). ACC/AHA versus ESC guidelines on heart failure: JACC guideline comparison. Journal of the American College of Cardiology, 73(21), 2756-2768. 10.1016/j.jacc.2019.03.478 31146820

[bibr45-08980101211018343] Wallström S. Ekman I. Omerovic E. Ulin K. Gyllensten H. (2019, March). Cohort study of healthcare use, costs and diagnoses from onset to 6 months after discharge for Takotsubo syndrome in Sweden. BMJ Open, 9(2), e027814. 10.1136/bmjopen-2018-027814 PMC6398620 30826802

[bibr46-08980101211018343] Wallström S. Ulin K. Määttä S. Omerovic E. Ekman I. (2016a, December). Impact of long-term stress in Takotsubo syndrome: Experience of patients. European Journal of Cardiovascular Nursing, 15(7), 522-528. 10.1177/1474515115618568 26572162 PMC5134193

[bibr47-08980101211018343] Wallström S. Ulin K. Omerovic E. Ekman I. (2016b, October). Symptoms in patients with Takotsubo syndrome: A qualitative interview study. BMJ Open, 6(10), e011820. 10.1136/bmjopen-2016-011820 PMC5073841 27707826

[bibr48-08980101211018343] Y-Hassan S. Tornvall P. (2018, February). Epidemiology, pathogenesis, and management of takotsubo syndrome. Clinical Autonomic Research: Official Journal of the Clinical Autonomic Research Society, 28(1), 53-65. 10.1007/s10286-017-0465-z 28917022 PMC5805795

[bibr49-08980101211018343] Yin R. K. (2009). Case study research: Design and methods. Sage Publications.

[bibr50-08980101211018343] Yoshikawa T. (2015, March). Takotsubo cardiomyopathy, a new concept of cardiomyopathy: Clinical features and pathophysiology. International Journal of Cardiology, 182, 297-303. 10.1016/j.ijcard.2014.12.116 25585367

